# Crystal structure of the non-steroidal anti-inflammatory drug (NSAID) tolmetin sodium

**DOI:** 10.1107/S2056989021000414

**Published:** 2021-01-19

**Authors:** Irina S. Konovalova, Sergiy M. Kovalenko, Dmitry V. Kravchenko, Vladimir P. Chuev

**Affiliations:** a SSI Institute for Single Crystals of the National Academy of Sciences of Ukraine, 61001, Kharkov, Ukraine; b V.N. Karazin Kharkiv National University, 4 Svobody Aq., Kharkiv, 61077, Ukraine; c Chemical Diversity Research Institute, 2A Rabochaya St, Khimki, Moscow Region, 141400, Russian Federation; d Federal State Autonomous Educational Institution of Higher Education, Belgorod State University, 85, Pobedy St, Belgorod, 308015, Russian Federation; e Experimental Plant for Dental Materials "VladmiVa", 81d, Michurin St, Belgorod, 308015, Russian Federation

**Keywords:** crystal structure, NSAIDs, sodium 2-(1-methyl-5-(4-methyl­benzo­yl)-1*H*-pyrrol-2-yl)acetate dihydrate, tolmetin sodium

## Abstract

The asymmetric unit of the title compound, sodium 2-[1-methyl-5-(4-methyl­benzo­yl)-1*H*-pyrrol-2-yl]acetate dihydrate, Na^+^·C_15_H_14_NO_3_^−^·2H_2_O, contains two sodium cations, two organic anions (*A* and *B*) and two water mol­ecules. The coordination geometry around the sodium cations corresponds to a distorted octa­hedron. Each pair of sodium cations (*A*–*A* or *B*–*B*) is chelated by two bridging anions coordinated by the O atoms of the deprotonated carb­oxy­lic groups, and each sodium atom is coordinated by an O atom of a third anion, which connects pairs of sodium atoms, and a water mol­ecule. As a result, a two-dimensional polymer is formed in the crystal. Hirshfeld surface analysis and two-dimensional fingerprint plots were used to analyze the inter­molecular contacts present in the crystal.

## Abstract 2

The asymmetric unit of the title two-dimensional polymer, sodium 2-[1-methyl-5-(4-methyl­benzo­yl)-1*H*-pyrrol-2-yl]acetate dihydrate, Na^+^·C_15_H_14_NO_3_^−^·2H_2_O, contains two sodium cations, two organic anions and two water mol­ecules. The title compound exhibits analgesic, anti-inflammatory and anti­pyretic activities.

## Chemical context   

Non-steroidal anti-inflammatory drugs (NSAIDs) are the gold standard for the management of acute or moderate pain associated with inflammatory changes or trauma (Klippel *et al.*, 2010 ▸). These drugs can suppress inflammation, lower body temperature, and reduce pain. In terms of the scale and frequency of use of NSAIDs, they rank first in the world. The combination of analgesic, anti-inflammatory and anti­pyretic effects determines the advantage of NSAIDs over other pain relievers. Tolmetin, which is one of the most widely used NSAIDs, belongs to the class of hetaryl­acetic acids (Moreland, 2004 ▸; McEvoy, 2007 ▸). It is commonly used for the treatment of rheumatoid arthritis, osteoarthritis, ankylosing spondylitis and periarticular disorders. Tolmetin sodium (CAS Number 64490-92-2) is the sodium salt form of tolmetin with analgesic, anti-inflammatory and anti­pyretic activities (Cordrey, 1976 ▸). In addition, the anti­cancer activity of Tolmetin has been studied and it was reported that tolmetin has effects on increasing the cytotoxic activity of anti-cancer drugs (Duffy *et al.*, 1998 ▸). It inhibits the function of β-catenin, so tolmetin can be used to develop new anti-cancer agents (Lu *et al.*, 2005 ▸).
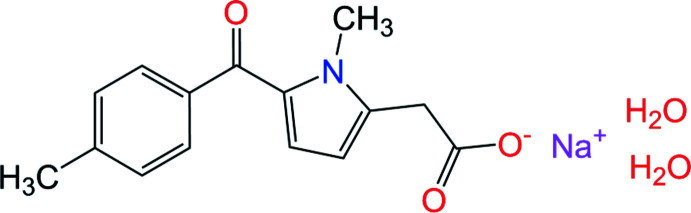


Recently, work has appeared on the use of this well-known active pharmaceutical compound tolmetin sodium for the development of new dosage forms, for example, novel rectal mucoadhesive hydro­gels (Ramadan *et al.*, 2018 ▸), thermosensitive mucoadhesive liquid suppositories for rectal delivery (Akl *et al.*, 2019 ▸) and different topical gel formulations (Auda *et al.*, 2015 ▸).

However, to date, the crystal structure of the substance tolmetin sodium has not been studied and described. Knowledge of the spatial structure of the crystal form of the active pharmaceutical compound is very important to ensure the quality and bioavailability of the drug and, according to the latest pharmacopoeia requirements, X-ray diffraction studies are mandatory for pharmaceutical development. In this work, we carried out an X-ray structural analysis of the crystal form of the substance tolmetin sodium and filled the gap in these studies.

## Structural commentary   

**Figure 1:**
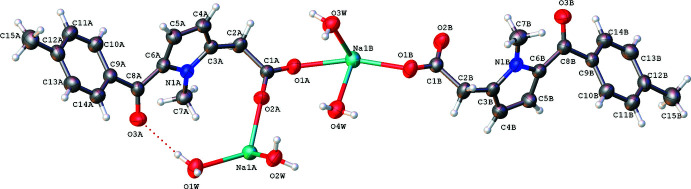
The mol­ecular structure of the title compound showing the atom-labeling scheme. Displacement ellipsoids are drawn at the 50% probability level.

**Figure 2:**
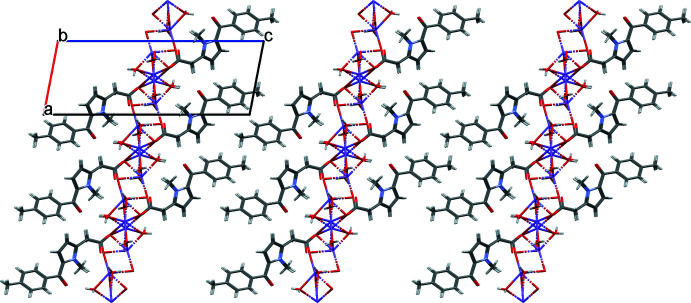
Two-dimensional polymeric chains in the crystal of the title compound.

The sodium salt of the C_15_H_14_NO_3_ organic anion exists in the crystal as a 1:2 hydrate (Fig. 1 ▸). The asymmetric unit contains two sodium cations, two organic anions (*A* and *B*) and two water mol­ecules. The coordination geometry around the sodium cations corresponds to a distorted octa­hedron. Each pair of sodium cations (*A*–*A* or *B*–*B*) is chelated by two bridging anions coordinated by the O atoms of the deprotonated carb­oxy­lic groups, and each sodium atom is coordinated by an O atom of a third anion, which connects pairs of sodium atoms, and a water mol­ecule. As a result, a two-dimensional polymer is formed in the crystal (Fig. 2 ▸). The Na—O_anion_ distances are 2.298 (2), 2.416 (2) and 2.441 (2) Å while the Na—O_water_ distances are on average slightly longer, being in the range 2.364 (2)–2.607 (3) Å. It is worth noting that the terminal atom O2*B* does not inter­act with a sodium cation.

The analysis of the mol­ecular structure of the anions showed that the terminal C1—O1 and C1—O2 bonds [1.249 (3) and 1.250 (3) Å in anion *A*, 1.243 (3) and 1.250 (3) Å in anion *B*] are very similar to each other and are slightly elongated in comparison with the standard value of 1.210 Å of a carbonyl group (Burgi *et al.*, 1994 ▸). It is also much shorter than the standard C—O single bond observed for a hydroxyl group (1.362 Å). We can assume that the negative charge is delocalized on both terminal O atoms for each anion.

The toluene substituent is in a synperiplanar conformation with respect to the C5—C6 bond of the pyrrole ring: the C5—C6—C8—C9 torsion angle is 26.9 (4)° in mol­ecule *A* and −29.0 (4)° in mol­ecule *B*. The relative orientation of the toluene ring with respect to the pyrrole ring, neither planar nor perpendicular, is given by the the C6—C8—C9—C10 torsion angle: 41.3 (4)° in mol­ecule *A* and −38.2 (4)° in mol­ecule *B*. Such an orientation mainly minimizes the inter­molecular H⋯H repulsions.

## Supra­molecular features   

In the crystal, O—H⋯O hydrogen bonds (Table 1 ▸) are formed between H atoms of the water mol­ecules (donors) and O atoms of the anions (acceptors), forming a two-dimensional network parallel to (001).

## Hirshfeld surface analysis   

**Figure 3:**
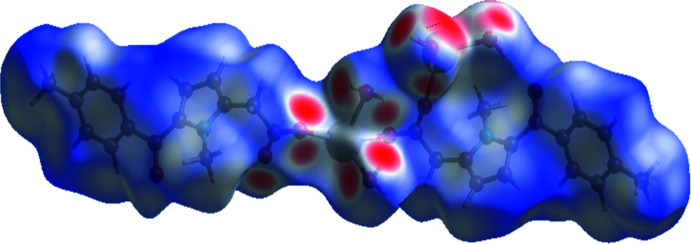
The Hirshfeld surface of the title compound mapped over *d*_norm_.

**Figure 4:**
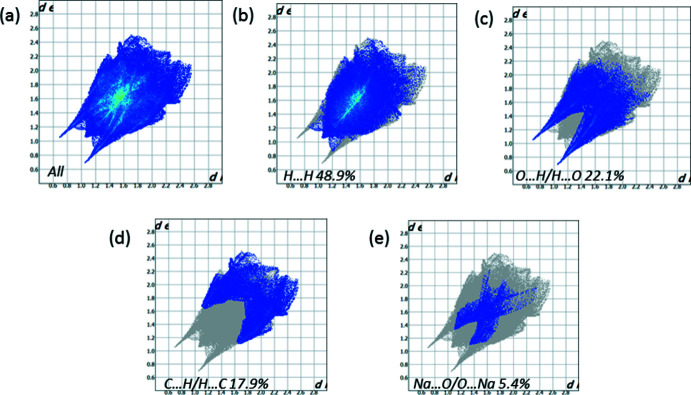
(*a*) The two-dimensional fingerprint plot for the title compound, and those delineated into (*b*) H⋯H (48.9%), (*c*) O⋯H/H⋯O (22.1%), (*d*) C⋯H/H⋯C (17.9%) and (*e*) Na⋯O/O⋯Na (5.4%) contacts.

*Crystal Explorer 17.5* (Turner *et al.*, 2017 ▸) was used to analyze the inter­actions in the crystal: fingerprint plots mapped over *d*_norm_ (Figs. 3 ▸ and 4 ▸) were generated. The mol­ecular Hirshfeld surfaces were obtained using a standard (high) surface resolution with the three-dimensional *d*_norm_ surfaces mapped over a fixed color scale of −0.666 (red) to 1.384 (blue). The areas colored red on the *d*_norm_-mapped Hirshfeld surfaces (Fig. 3 ▸) correspond to the contacts which are shorter than van der Waals radii sum of the closest atoms. As can be seen in Fig. 4 ▸, short contacts are present at the hydrogen atoms and oxygen lone pair of the water mol­ecules. In addition, the areas of short contacts are located at the oxygen atoms of carbonyl groups (Fig. 3 ▸).

All the inter­molecular inter­actions of the title compound are shown in the two-dimensional fingerprint plot presented in Fig. 4 ▸. The contribution of the O⋯H/H⋯O contacts, corresponding to the O—H⋯O inter­action, is represented by a pair of long sharp spikes (22.1%). This indicates that O—H⋯O hydrogen bonds are the strongest inter­actions in the crystal of the title compound (Fig. 4 ▸).

## Database survey   

A search of the Cambridge Structural Database (CSD, Version 5.41, update of November 2019; Groom *et al.*, 2016 ▸) for the 2-(1-methyl-5-(4-methyl­benzo­yl)-1*H*-pyrrol-2-yl)acetate skeleton yielded only two hits, 2-meth­oxy­phenyl 2-{2-[1-methyl-5-(4-methyl­benzo­yl)pyrrol-2-yl]acetamido}­acetate (CSD refcode MODNID; Lou *et al.*, 2008 ▸) and bis­(di­methyl­sulfoxide-*O*)tetra­kis­[μ_2_-1-methyl-5-(*p*-toluo­yl)-1*H*-pyrrole-2-acetato-*O*,*O*′]dicopper(II) (SETBIC; Dendrinou-Samara *et al.*, 1990 ▸).

## Crystallization   

**Figure 5:**
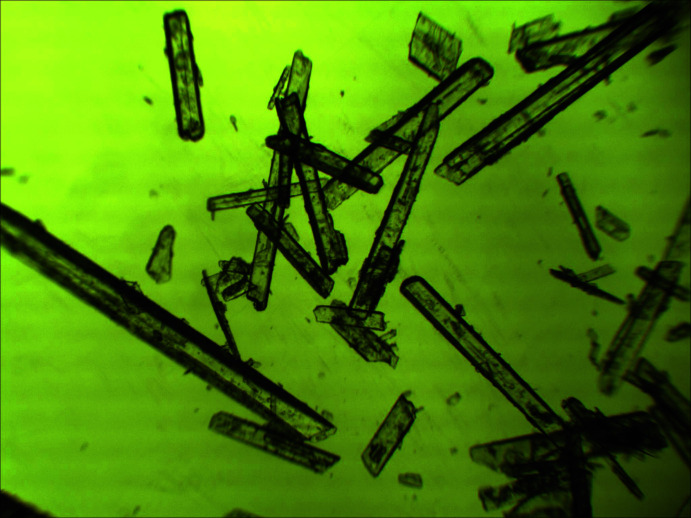
Crystals of tolmetin sodium.

Crystallization by slow evaporation of an aqueous solution of tolmetin sodium was carried out to provide colorless block-shaped single crystals suitable for a X-ray diffraction analysis (Fig. 5 ▸).

## Refinement   

Crystal data, data collection and structure refinement details are summarized in Table 2 ▸. H atoms were placed in calculated positions (O—H = 0.98 Å, C—H = 0.93–0.96 Å) and refined as riding with *U*_iso_H = 1.2*U*_eq_(C) or 1.5*U*_eq_(O, C-meth­yl).

## Supplementary Material

Crystal structure: contains datablock(s) I. DOI: 10.1107/S2056989021000414/zq2259sup1.cif

Structure factors: contains datablock(s) I. DOI: 10.1107/S2056989021000414/zq2259Isup2.hkl

CCDC reference: 2055407

Additional supporting information:  crystallographic information; 3D view; checkCIF report

## Figures and Tables

**Table 1:** Hydrogen-bond geometry (Å, °)

*D*—H⋯*A*	*D*—H	H⋯*A*	*D*⋯*A*	*D*—H⋯*A*
O1*W*—H1*WA*⋯O3*A*	0.89	2.12	3.002 (3)	169
O1*W*—H1*WB*⋯O1*A* ^i^	0.89	2.00	2.665 (4)	130
O2*W*—H2*WA*⋯O2*B* ^i^	0.89	1.88	2.741 (3)	161
O2*W*—H2*WB*⋯O2*B* ^ii^	0.89	2.13	3.019 (3)	172
O3*W*—H3*WA*⋯O3*B* ^iii^	0.89	2.16	2.961 (3)	150
O3*W*—H3*WB*⋯O1*B* ^ii^	0.89	1.84	2.699 (4)	161
O4*W*—H4*WA*⋯O1*B* ^ii^	0.89	2.19	2.894 (3)	136
O4*W*—H4*WB*⋯O3*W* ^ii^	0.89	2.02	2.889 (3)	163

Symmetry codes: (i) 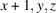; (ii) 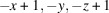; (iii) 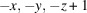.

**Table 2:** Experimental details

Crystal data	
Chemical formula	2Na^+^·2C_15_H_14_NO_3_^−^·4H_2_O
*M*_r_	630.59
Crystal system, space group	Triclinic, *P* 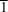
Temperature (K)	293
*a*, *b*, *c* (Å)	8.5404 (8), 9.0144 (9), 21.6217 (19)
α, β, γ (°)	92.922 (8), 98.873 (7), 113.038 (9)
*V* (Å^3^)	1502.1 (3)
*Z*	2
Radiation type	Mo *K*α
μ (mm^−1^)	0.13
Crystal size (mm)	0.4 × 0.2 × 0.1

Data collection	
Diffractometer	Rigaku Oxford Diffraction Xcalibur, Sapphire3
Absorption correction	Multi-scan (*CrysAlis PRO*; Rigaku OD, 2018 ▸)
*T*_min_, *T*_max_	0.737, 1.000
No. of measured, independent and observed [*I* > 2σ(*I*)] reflections	11985, 5283, 3288
*R*_int_	0.060
(sin θ/λ)_max_ (Å^−1^)	0.595

Refinement	
*R*[*F* ^2^ > 2σ(*F* ^2^)], *wR*(*F* ^2^), *S*	0.064, 0.191, 1.00
No. of reflections	5283
No. of parameters	403
H-atom treatment	H-atom parameters constrained
Δρ_max_, Δρ_min_ (e Å^−3^)	0.38, −0.28

Computer programs: *CrysAlis PRO* (Rigaku OD, 2018 ▸), *SHELXT* (Sheldrick, 2015*a* ▸), *SHELXL* (Sheldrick, 2015*b* ▸) and *OLEX2* (Dolomanov *et al.*, 2009 ▸).

## supplementary crystallographic information

### Crystal data

**Table:**

2Na^+^·2C_15_H_14_NO_3_^−^·4H_2_O	*Z* = 2
*M**_r_* = 630.59	*F*(000) = 664
Triclinic, *P*1	*D*_x_ = 1.394 Mg m^−^^3^
*a* = 8.5404 (8) Å	Mo *K*α radiation, λ = 0.71073 Å
*b* = 9.0144 (9) Å	Cell parameters from 1420 reflections
*c* = 21.6217 (19) Å	θ = 3.1–24.2°
α = 92.922 (8)°	µ = 0.13 mm^−^^1^
β = 98.873 (7)°	*T* = 293 K
γ = 113.038 (9)°	Block, colourless
*V* = 1502.1 (3) Å^3^	0.4 × 0.2 × 0.1 mm

### Data collection

**Table:**

Rigaku Oxford Diffraction Xcalibur, Sapphire3 diffractometer	5283 independent reflections
Radiation source: fine-focus sealed X-ray tube, Enhance (Mo) X-ray Source	3288 reflections with *I* > 2σ(*I*)
Graphite monochromator	*R*_int_ = 0.060
Detector resolution: 16.1827 pixels mm^-1^	θ_max_ = 25.0°, θ_min_ = 2.9°
phi and ω scans	*h* = −10→10
Absorption correction: multi-scan (CrysAlisPro; Rigaku OD, 2018)	*k* = −10→10
*T*_min_ = 0.737, *T*_max_ = 1.000	*l* = −25→25
11985 measured reflections	

### Refinement

**Table:**

Refinement on *F*^2^	Primary atom site location: inferred from neighbouring sites
Least-squares matrix: full	Hydrogen site location: difference Fourier map
*R*[*F*^2^ > 2σ(*F*^2^)] = 0.064	H-atom parameters constrained
*wR*(*F*^2^) = 0.191	*w* = 1/[σ^2^(*F*_o_^2^) + (0.0844*P*)^2^] where *P* = (*F*_o_^2^ + 2*F*_c_^2^)/3
*S* = 1.00	(Δ/σ)_max_ < 0.001
5283 reflections	Δρ_max_ = 0.38 e Å^−^^3^
403 parameters	Δρ_min_ = −0.28 e Å^−^^3^
0 restraints	

### Special details

**Table:**

Geometry. All esds (except the esd in the dihedral angle between two l.s. planes) are estimated using the full covariance matrix. The cell esds are taken into account individually in the estimation of esds in distances, angles and torsion angles; correlations between esds in cell parameters are only used when they are defined by crystal symmetry. An approximate (isotropic) treatment of cell esds is used for estimating esds involving l.s. planes.

### Fractional atomic coordinates and isotropic or equivalent isotropic displacement parameters (Å^2^)

**Table:**

	*x*	*y*	*z*	*U*_iso_*/*U*_eq_	
Na1A	1.15418 (14)	0.48055 (14)	0.46665 (5)	0.0463 (4)	
O1A	0.6328 (3)	0.4448 (3)	0.43538 (9)	0.0463 (5)	
O2A	0.8756 (3)	0.4310 (3)	0.41918 (9)	0.0527 (6)	
O3A	1.3144 (3)	0.5970 (3)	0.27170 (10)	0.0572 (6)	
N1A	0.9595 (3)	0.5322 (3)	0.28684 (10)	0.0370 (6)	
C1A	0.7367 (4)	0.4452 (3)	0.40052 (13)	0.0369 (7)	
C2A	0.6907 (4)	0.4728 (4)	0.33229 (13)	0.0441 (7)	
H2AA	0.568753	0.405386	0.316973	0.053*	
H2AB	0.705297	0.584995	0.332112	0.053*	
C3A	0.7899 (4)	0.4394 (4)	0.28655 (13)	0.0397 (7)	
C4A	0.7319 (4)	0.3165 (4)	0.23764 (14)	0.0445 (7)	
H4A	0.620599	0.234969	0.227154	0.053*	
C5A	0.8671 (4)	0.3346 (4)	0.20653 (14)	0.0446 (7)	
H5A	0.862808	0.267733	0.171513	0.053*	
C6A	1.0108 (4)	0.4710 (4)	0.23717 (13)	0.0403 (7)	
C7A	1.0633 (4)	0.6845 (3)	0.32769 (13)	0.0453 (7)	
H7AA	1.124646	0.663236	0.364871	0.068*	
H7AB	0.988702	0.733150	0.339497	0.068*	
H7AC	1.144888	0.757182	0.305536	0.068*	
C8A	1.1889 (4)	0.5209 (4)	0.22929 (13)	0.0392 (7)	
C9A	1.2180 (4)	0.4679 (3)	0.16703 (13)	0.0399 (7)	
C10A	1.1169 (4)	0.4703 (4)	0.11027 (14)	0.0457 (7)	
H10A	1.026896	0.503734	0.110630	0.055*	
C11A	1.1493 (4)	0.4236 (4)	0.05367 (14)	0.0488 (8)	
H11A	1.079818	0.425219	0.016307	0.059*	
C12A	1.2836 (4)	0.3741 (4)	0.05106 (15)	0.0455 (8)	
C13A	1.3832 (4)	0.3726 (4)	0.10725 (15)	0.0508 (8)	
H13A	1.472156	0.337798	0.106762	0.061*	
C14A	1.3541 (4)	0.4217 (4)	0.16450 (15)	0.0478 (8)	
H14A	1.426516	0.423803	0.201665	0.057*	
C15A	1.3099 (5)	0.3182 (4)	−0.01169 (16)	0.0620 (9)	
H15A	1.253192	0.201892	−0.019774	0.093*	
H15B	1.431599	0.351811	−0.011128	0.093*	
H15C	1.261676	0.365083	−0.044267	0.093*	
Na1B	0.45480 (15)	0.23476 (14)	0.49496 (5)	0.0497 (4)	
O1B	0.3539 (3)	0.0551 (3)	0.57285 (10)	0.0520 (6)	
O2B	0.1279 (3)	0.0952 (3)	0.59464 (9)	0.0517 (6)	
O3B	−0.3202 (3)	−0.0817 (3)	0.73382 (10)	0.0561 (6)	
N1B	0.0316 (3)	−0.0303 (3)	0.72107 (10)	0.0392 (6)	
C1B	0.2576 (4)	0.0630 (3)	0.60931 (13)	0.0372 (7)	
C2B	0.2993 (4)	0.0232 (4)	0.67578 (13)	0.0469 (8)	
H2BA	0.277121	−0.091173	0.673239	0.056*	
H2BB	0.422204	0.084105	0.691795	0.056*	
C3B	0.2033 (4)	0.0571 (4)	0.72214 (13)	0.0405 (7)	
C4B	0.2635 (4)	0.1815 (4)	0.77089 (14)	0.0505 (8)	
H4B	0.375714	0.261529	0.781418	0.061*	
C5B	0.1287 (4)	0.1671 (4)	0.80151 (14)	0.0451 (7)	
H5B	0.134436	0.234532	0.836595	0.054*	
C6B	−0.0169 (4)	0.0342 (4)	0.77058 (13)	0.0414 (7)	
C7B	−0.0783 (4)	−0.1782 (4)	0.67844 (14)	0.0505 (8)	
H7BA	−0.140894	−0.151452	0.643088	0.076*	
H7BB	−0.158712	−0.252622	0.700442	0.076*	
H7BC	−0.007306	−0.227504	0.663767	0.076*	
C8B	−0.1949 (4)	−0.0101 (4)	0.77748 (14)	0.0423 (7)	
C9B	−0.2254 (4)	0.0398 (4)	0.83992 (13)	0.0396 (7)	
C10B	−0.1236 (4)	0.0352 (4)	0.89619 (14)	0.0458 (8)	
H10B	−0.034555	0.000647	0.895357	0.055*	
C11B	−0.1552 (4)	0.0819 (4)	0.95293 (14)	0.0483 (8)	
H11B	−0.086237	0.078848	0.990196	0.058*	
C12B	−0.2879 (4)	0.1337 (4)	0.95586 (14)	0.0463 (8)	
C13B	−0.3882 (4)	0.1359 (4)	0.89975 (15)	0.0505 (8)	
H13B	−0.477015	0.170816	0.900353	0.061*	
C14B	−0.3588 (4)	0.0871 (4)	0.84272 (15)	0.0473 (8)	
H14B	−0.430573	0.086230	0.805569	0.057*	
C15B	−0.3154 (4)	0.1885 (5)	1.01896 (16)	0.0610 (9)	
H15D	−0.263611	0.144401	1.051608	0.091*	
H15E	−0.437342	0.150939	1.018731	0.091*	
H15F	−0.262690	0.304934	1.026643	0.091*	
O1W	1.3788 (3)	0.5461 (3)	0.40784 (10)	0.0541 (6)	
H1WA	1.363692	0.575550	0.369478	0.081*	
H1WB	1.405922	0.460320	0.406558	0.081*	
O2W	1.1457 (3)	0.2133 (3)	0.48044 (9)	0.0529 (6)	
H2WA	1.128262	0.190038	0.518967	0.079*	
H2WB	1.066822	0.127148	0.455027	0.079*	
O3W	0.3857 (3)	0.0443 (3)	0.40179 (10)	0.0522 (6)	
H3WA	0.383694	0.094075	0.367336	0.078*	
H3WB	0.464794	0.002995	0.401246	0.078*	
O4W	0.7371 (3)	0.2175 (3)	0.52238 (10)	0.0538 (6)	
H4WA	0.769350	0.168269	0.493211	0.081*	
H4WB	0.720981	0.138360	0.546861	0.081*	

### Atomic displacement parameters (Å^2^)

**Table:**

	*U*^11^	*U*^22^	*U*^33^	*U*^12^	*U*^13^	*U*^23^
Na1A	0.0365 (7)	0.0608 (8)	0.0418 (7)	0.0197 (6)	0.0075 (5)	0.0091 (6)
O1A	0.0426 (12)	0.0665 (14)	0.0355 (12)	0.0258 (11)	0.0121 (9)	0.0097 (10)
O2A	0.0335 (12)	0.0802 (16)	0.0450 (13)	0.0235 (11)	0.0045 (9)	0.0188 (11)
O3A	0.0405 (13)	0.0796 (17)	0.0398 (13)	0.0148 (12)	0.0031 (10)	−0.0030 (11)
N1A	0.0369 (14)	0.0414 (13)	0.0326 (13)	0.0153 (11)	0.0071 (10)	0.0057 (10)
C1A	0.0346 (16)	0.0406 (16)	0.0358 (16)	0.0154 (13)	0.0062 (13)	0.0090 (13)
C2A	0.0430 (17)	0.0605 (19)	0.0377 (17)	0.0287 (16)	0.0091 (13)	0.0145 (14)
C3A	0.0360 (16)	0.0535 (18)	0.0337 (16)	0.0211 (14)	0.0083 (12)	0.0098 (13)
C4A	0.0381 (17)	0.0456 (18)	0.0409 (17)	0.0093 (14)	0.0030 (13)	0.0040 (14)
C5A	0.0436 (18)	0.0441 (17)	0.0417 (17)	0.0138 (15)	0.0081 (14)	0.0008 (14)
C6A	0.0422 (17)	0.0475 (17)	0.0310 (16)	0.0164 (14)	0.0105 (13)	0.0049 (13)
C7A	0.0509 (19)	0.0416 (16)	0.0377 (17)	0.0149 (15)	0.0046 (14)	−0.0007 (13)
C8A	0.0367 (16)	0.0446 (17)	0.0377 (17)	0.0158 (14)	0.0115 (13)	0.0092 (13)
C9A	0.0382 (17)	0.0432 (17)	0.0344 (16)	0.0121 (14)	0.0090 (12)	0.0016 (13)
C10A	0.0449 (18)	0.0544 (19)	0.0398 (18)	0.0215 (16)	0.0087 (14)	0.0085 (15)
C11A	0.052 (2)	0.0553 (19)	0.0364 (17)	0.0195 (17)	0.0064 (14)	0.0068 (14)
C12A	0.0412 (18)	0.0457 (18)	0.0473 (19)	0.0129 (15)	0.0147 (14)	0.0052 (14)
C13A	0.0431 (18)	0.063 (2)	0.050 (2)	0.0244 (17)	0.0117 (15)	0.0034 (16)
C14A	0.0442 (18)	0.059 (2)	0.0394 (17)	0.0204 (16)	0.0067 (14)	0.0085 (15)
C15A	0.061 (2)	0.067 (2)	0.055 (2)	0.0201 (19)	0.0208 (17)	−0.0028 (17)
Na1B	0.0459 (7)	0.0559 (8)	0.0458 (7)	0.0174 (6)	0.0125 (5)	0.0075 (6)
O1B	0.0549 (14)	0.0749 (16)	0.0407 (12)	0.0366 (12)	0.0200 (10)	0.0163 (11)
O2B	0.0505 (13)	0.0781 (16)	0.0399 (12)	0.0372 (12)	0.0140 (10)	0.0148 (11)
O3B	0.0418 (13)	0.0753 (16)	0.0411 (13)	0.0154 (12)	0.0042 (10)	−0.0022 (11)
N1B	0.0411 (14)	0.0489 (14)	0.0304 (13)	0.0200 (12)	0.0092 (10)	0.0062 (11)
C1B	0.0401 (17)	0.0379 (15)	0.0334 (16)	0.0152 (13)	0.0087 (13)	0.0024 (12)
C2B	0.0497 (19)	0.063 (2)	0.0358 (17)	0.0279 (16)	0.0147 (14)	0.0139 (15)
C3B	0.0379 (17)	0.0560 (19)	0.0327 (16)	0.0228 (15)	0.0081 (12)	0.0129 (14)
C4B	0.0376 (17)	0.061 (2)	0.0451 (19)	0.0123 (16)	0.0061 (14)	0.0113 (16)
C5B	0.0419 (17)	0.0538 (19)	0.0346 (17)	0.0148 (15)	0.0076 (13)	−0.0031 (14)
C6B	0.0379 (17)	0.0523 (18)	0.0322 (16)	0.0162 (15)	0.0061 (12)	0.0073 (13)
C7B	0.056 (2)	0.0468 (18)	0.0443 (19)	0.0197 (16)	0.0020 (15)	−0.0011 (14)
C8B	0.0414 (18)	0.0483 (18)	0.0370 (17)	0.0169 (15)	0.0093 (14)	0.0086 (14)
C9B	0.0345 (16)	0.0461 (17)	0.0373 (17)	0.0137 (14)	0.0104 (12)	0.0083 (13)
C10B	0.0464 (18)	0.0572 (19)	0.0385 (17)	0.0252 (16)	0.0092 (14)	0.0086 (14)
C11B	0.0488 (19)	0.061 (2)	0.0369 (18)	0.0231 (17)	0.0097 (14)	0.0085 (15)
C12B	0.0432 (18)	0.0501 (18)	0.0411 (18)	0.0109 (15)	0.0171 (14)	0.0046 (14)
C13B	0.0414 (18)	0.059 (2)	0.053 (2)	0.0226 (16)	0.0099 (15)	0.0015 (16)
C14B	0.0420 (18)	0.0561 (19)	0.0426 (18)	0.0192 (16)	0.0063 (14)	0.0067 (15)
C15B	0.055 (2)	0.074 (2)	0.052 (2)	0.0217 (19)	0.0174 (16)	−0.0008 (17)
O1W	0.0640 (15)	0.0639 (14)	0.0506 (13)	0.0369 (12)	0.0237 (11)	0.0180 (11)
O2W	0.0596 (14)	0.0589 (14)	0.0377 (12)	0.0211 (12)	0.0085 (10)	0.0093 (10)
O3W	0.0558 (14)	0.0637 (15)	0.0429 (12)	0.0306 (12)	0.0092 (10)	0.0048 (11)
O4W	0.0626 (15)	0.0585 (14)	0.0422 (12)	0.0260 (12)	0.0129 (10)	0.0023 (10)

### Geometric parameters (Å, º)

**Table:**

Na1A—Na1A^i^	3.314 (2)	Na1B—O1W^i^	2.607 (3)
Na1A—O1A^i^	2.441 (2)	Na1B—O2W^iii^	2.538 (2)
Na1A—O2A	2.298 (2)	Na1B—O3W	2.417 (2)
Na1A—O2A^i^	2.636 (2)	Na1B—O4W	2.460 (2)
Na1A—C1A^i^	2.838 (3)	O1B—C1B	1.243 (3)
Na1A—Na1B^ii^	3.9976 (16)	O2B—C1B	1.250 (3)
Na1A—Na1B^i^	3.2629 (17)	O3B—C8B	1.241 (4)
Na1A—O1W	2.364 (2)	N1B—C3B	1.362 (4)
Na1A—O2W	2.417 (2)	N1B—C6B	1.385 (4)
Na1A—O4W^i^	2.493 (2)	N1B—C7B	1.461 (4)
O1A—C1A	1.249 (3)	C1B—C2B	1.522 (4)
O1A—Na1B	2.481 (2)	C2B—H2BA	0.9700
O2A—C1A	1.250 (3)	C2B—H2BB	0.9700
O3A—C8A	1.232 (3)	C2B—C3B	1.479 (4)
N1A—C3A	1.359 (4)	C3B—C4B	1.376 (4)
N1A—C6A	1.387 (3)	C4B—H4B	0.9300
N1A—C7A	1.461 (4)	C4B—C5B	1.382 (4)
C1A—C2A	1.526 (4)	C5B—H5B	0.9300
C2A—H2AA	0.9700	C5B—C6B	1.387 (4)
C2A—H2AB	0.9700	C6B—C8B	1.446 (4)
C2A—C3A	1.488 (4)	C7B—H7BA	0.9600
C3A—C4A	1.371 (4)	C7B—H7BB	0.9600
C4A—H4A	0.9300	C7B—H7BC	0.9600
C4A—C5A	1.384 (4)	C8B—C9B	1.494 (4)
C5A—H5A	0.9300	C9B—C10B	1.395 (4)
C5A—C6A	1.394 (4)	C9B—C14B	1.371 (4)
C6A—C8A	1.448 (4)	C10B—H10B	0.9300
C7A—H7AA	0.9600	C10B—C11B	1.376 (4)
C7A—H7AB	0.9600	C11B—H11B	0.9300
C7A—H7AC	0.9600	C11B—C12B	1.393 (4)
C8A—C9A	1.494 (4)	C12B—C13B	1.379 (4)
C9A—C10A	1.394 (4)	C12B—C15B	1.510 (4)
C9A—C14A	1.387 (4)	C13B—H13B	0.9300
C10A—H10A	0.9300	C13B—C14B	1.380 (4)
C10A—C11A	1.376 (4)	C14B—H14B	0.9300
C11A—H11A	0.9300	C15B—H15D	0.9600
C11A—C12A	1.391 (4)	C15B—H15E	0.9600
C12A—C13A	1.375 (4)	C15B—H15F	0.9600
C12A—C15A	1.503 (4)	O1W—H1WA	0.8897
C13A—H13A	0.9300	O1W—H1WB	0.8899
C13A—C14A	1.384 (4)	O2W—H2WA	0.8899
C14A—H14A	0.9300	O2W—H2WB	0.8899
C15A—H15A	0.9600	O3W—H3WA	0.8905
C15A—H15B	0.9600	O3W—H3WB	0.8905
C15A—H15C	0.9600	O4W—H4WA	0.8897
Na1B—O1B	2.416 (2)	O4W—H4WB	0.8902
			
Na1A^i^—Na1A—Na1B^ii^	135.58 (5)	H15A—C15A—H15C	109.5
O1A^i^—Na1A—Na1A^i^	94.89 (7)	H15B—C15A—H15C	109.5
O1A^i^—Na1A—O2A^i^	51.31 (6)	Na1A^i^—Na1B—Na1A^iii^	103.67 (4)
O1A^i^—Na1A—C1A^i^	26.00 (7)	O1A—Na1B—Na1A^i^	47.95 (5)
O1A^i^—Na1A—Na1B^i^	49.01 (5)	O1A—Na1B—Na1A^iii^	81.27 (6)
O1A^i^—Na1A—Na1B^ii^	61.96 (6)	O1A—Na1B—O1W^i^	83.94 (8)
O1A^i^—Na1A—O4W^i^	78.93 (8)	O1A—Na1B—O2W^iii^	111.77 (8)
O2A^i^—Na1A—Na1A^i^	43.62 (5)	O1B—Na1B—Na1A^i^	116.87 (7)
O2A—Na1A—Na1A^i^	52.29 (6)	O1B—Na1B—Na1A^iii^	107.75 (6)
O2A—Na1A—O1A^i^	147.11 (9)	O1B—Na1B—O1A	164.64 (8)
O2A—Na1A—O2A^i^	95.91 (8)	O1B—Na1B—O1W^i^	84.57 (8)
O2A^i^—Na1A—C1A^i^	26.07 (7)	O1B—Na1B—O2W^iii^	81.05 (7)
O2A—Na1A—C1A^i^	121.17 (9)	O1B—Na1B—O3W	101.75 (9)
O2A^i^—Na1A—Na1B^ii^	104.45 (6)	O1B—Na1B—O4W	88.42 (8)
O2A—Na1A—Na1B^i^	142.94 (8)	O1W^i^—Na1B—Na1A^i^	45.82 (5)
O2A^i^—Na1A—Na1B^i^	84.23 (6)	O1W^i^—Na1B—Na1A^iii^	84.57 (6)
O2A—Na1A—Na1B^ii^	138.04 (8)	O2W^iii^—Na1B—Na1A^iii^	35.20 (5)
O2A—Na1A—O1W	121.00 (9)	O2W^iii^—Na1B—Na1A^i^	137.63 (7)
O2A—Na1A—O2W	103.90 (9)	O2W^iii^—Na1B—O1W^i^	104.60 (8)
O2A—Na1A—O4W^i^	95.91 (8)	O3W—Na1B—Na1A^iii^	107.78 (6)
C1A^i^—Na1A—Na1A^i^	69.18 (7)	O3W—Na1B—Na1A^i^	118.49 (7)
C1A^i^—Na1A—Na1B^ii^	80.46 (6)	O3W—Na1B—O1A	86.73 (8)
C1A^i^—Na1A—Na1B^i^	68.45 (6)	O3W—Na1B—O1W^i^	163.22 (8)
Na1B^i^—Na1A—Na1A^i^	118.26 (6)	O3W—Na1B—O2W^iii^	91.80 (8)
Na1B^i^—Na1A—Na1B^ii^	76.33 (4)	O3W—Na1B—O4W	89.29 (8)
O1W—Na1A—Na1A^i^	158.68 (9)	O4W—Na1B—Na1A^iii^	152.90 (7)
O1W—Na1A—O1A^i^	90.21 (8)	O4W—Na1B—Na1A^i^	49.23 (6)
O1W—Na1A—O2A^i^	136.23 (9)	O4W—Na1B—O1A	78.81 (8)
O1W—Na1A—C1A^i^	115.58 (9)	O4W—Na1B—O1W^i^	75.24 (8)
O1W—Na1A—Na1B^i^	52.29 (6)	O4W—Na1B—O2W^iii^	169.41 (8)
O1W—Na1A—Na1B^ii^	64.50 (6)	C1B—O1B—Na1B	126.86 (19)
O1W—Na1A—O2W	93.84 (8)	C3B—N1B—C6B	109.0 (2)
O1W—Na1A—O4W^i^	79.14 (8)	C3B—N1B—C7B	125.0 (2)
O2W—Na1A—Na1A^i^	107.35 (7)	C6B—N1B—C7B	125.7 (2)
O2W—Na1A—O1A^i^	82.17 (8)	O1B—C1B—O2B	125.0 (3)
O2W—Na1A—O2A^i^	99.52 (8)	O1B—C1B—C2B	116.3 (3)
O2W—Na1A—C1A^i^	86.62 (8)	O2B—C1B—C2B	118.7 (2)
O2W—Na1A—Na1B^i^	112.65 (7)	C1B—C2B—H2BA	108.2
O2W—Na1A—Na1B^ii^	37.26 (6)	C1B—C2B—H2BB	108.2
O2W—Na1A—O4W^i^	159.77 (8)	H2BA—C2B—H2BB	107.3
O4W^i^—Na1A—Na1A^i^	81.55 (7)	C3B—C2B—C1B	116.5 (2)
O4W^i^—Na1A—O2A^i^	74.06 (8)	C3B—C2B—H2BA	108.2
O4W^i^—Na1A—C1A^i^	79.57 (8)	C3B—C2B—H2BB	108.2
O4W^i^—Na1A—Na1B^i^	48.35 (6)	N1B—C3B—C2B	123.9 (3)
O4W^i^—Na1A—Na1B^ii^	124.68 (6)	N1B—C3B—C4B	107.9 (2)
Na1A^i^—O1A—Na1B	83.04 (7)	C4B—C3B—C2B	128.1 (3)
C1A—O1A—Na1A^i^	95.05 (17)	C3B—C4B—H4B	125.9
C1A—O1A—Na1B	132.45 (19)	C3B—C4B—C5B	108.2 (3)
Na1A—O2A—Na1A^i^	84.09 (8)	C5B—C4B—H4B	125.9
C1A—O2A—Na1A^i^	86.05 (17)	C4B—C5B—H5B	126.1
C1A—O2A—Na1A	163.4 (2)	C4B—C5B—C6B	107.8 (3)
C3A—N1A—C6A	109.3 (2)	C6B—C5B—H5B	126.1
C3A—N1A—C7A	124.8 (2)	N1B—C6B—C5B	107.0 (3)
C6A—N1A—C7A	125.4 (2)	N1B—C6B—C8B	123.9 (3)
O1A—C1A—Na1A^i^	58.95 (14)	C5B—C6B—C8B	127.8 (3)
O1A—C1A—O2A	123.8 (3)	N1B—C7B—H7BA	109.5
O1A—C1A—C2A	116.3 (2)	N1B—C7B—H7BB	109.5
O2A—C1A—Na1A^i^	67.89 (15)	N1B—C7B—H7BC	109.5
O2A—C1A—C2A	119.8 (2)	H7BA—C7B—H7BB	109.5
C2A—C1A—Na1A^i^	158.3 (2)	H7BA—C7B—H7BC	109.5
C1A—C2A—H2AA	107.9	H7BB—C7B—H7BC	109.5
C1A—C2A—H2AB	107.9	O3B—C8B—C6B	123.2 (3)
H2AA—C2A—H2AB	107.2	O3B—C8B—C9B	119.5 (3)
C3A—C2A—C1A	117.4 (2)	C6B—C8B—C9B	117.3 (3)
C3A—C2A—H2AA	107.9	C10B—C9B—C8B	121.3 (3)
C3A—C2A—H2AB	107.9	C14B—C9B—C8B	120.0 (3)
N1A—C3A—C2A	123.8 (3)	C14B—C9B—C10B	118.7 (3)
N1A—C3A—C4A	108.1 (2)	C9B—C10B—H10B	120.1
C4A—C3A—C2A	128.1 (3)	C11B—C10B—C9B	119.9 (3)
C3A—C4A—H4A	125.8	C11B—C10B—H10B	120.1
C3A—C4A—C5A	108.4 (3)	C10B—C11B—H11B	119.2
C5A—C4A—H4A	125.8	C10B—C11B—C12B	121.5 (3)
C4A—C5A—H5A	126.2	C12B—C11B—H11B	119.2
C4A—C5A—C6A	107.6 (3)	C11B—C12B—C15B	120.0 (3)
C6A—C5A—H5A	126.2	C13B—C12B—C11B	117.7 (3)
N1A—C6A—C5A	106.6 (3)	C13B—C12B—C15B	122.3 (3)
N1A—C6A—C8A	124.4 (3)	C12B—C13B—H13B	119.5
C5A—C6A—C8A	127.6 (3)	C12B—C13B—C14B	121.1 (3)
N1A—C7A—H7AA	109.5	C14B—C13B—H13B	119.5
N1A—C7A—H7AB	109.5	C9B—C14B—C13B	121.1 (3)
N1A—C7A—H7AC	109.5	C9B—C14B—H14B	119.5
H7AA—C7A—H7AB	109.5	C13B—C14B—H14B	119.5
H7AA—C7A—H7AC	109.5	C12B—C15B—H15D	109.5
H7AB—C7A—H7AC	109.5	C12B—C15B—H15E	109.5
O3A—C8A—C6A	123.4 (3)	C12B—C15B—H15F	109.5
O3A—C8A—C9A	119.5 (3)	H15D—C15B—H15E	109.5
C6A—C8A—C9A	117.0 (3)	H15D—C15B—H15F	109.5
C10A—C9A—C8A	122.0 (3)	H15E—C15B—H15F	109.5
C14A—C9A—C8A	119.9 (3)	Na1A—O1W—Na1B^i^	81.89 (7)
C14A—C9A—C10A	118.0 (3)	Na1A—O1W—H1WA	120.2
C9A—C10A—H10A	119.7	Na1A—O1W—H1WB	105.3
C11A—C10A—C9A	120.5 (3)	Na1B^i^—O1W—H1WA	120.3
C11A—C10A—H10A	119.7	Na1B^i^—O1W—H1WB	115.2
C10A—C11A—H11A	119.2	H1WA—O1W—H1WB	110.6
C10A—C11A—C12A	121.5 (3)	Na1A—O2W—Na1B^ii^	107.54 (9)
C12A—C11A—H11A	119.2	Na1A—O2W—H2WA	110.1
C11A—C12A—C15A	119.7 (3)	Na1A—O2W—H2WB	119.8
C13A—C12A—C11A	117.6 (3)	Na1B^ii^—O2W—H2WA	97.1
C13A—C12A—C15A	122.7 (3)	Na1B^ii^—O2W—H2WB	115.5
C12A—C13A—H13A	119.2	H2WA—O2W—H2WB	104.2
C12A—C13A—C14A	121.5 (3)	Na1B—O3W—H3WA	110.1
C14A—C13A—H13A	119.2	Na1B—O3W—H3WB	111.9
C9A—C14A—H14A	119.7	H3WA—O3W—H3WB	106.7
C13A—C14A—C9A	120.7 (3)	Na1A^i^—O4W—H4WA	118.6
C13A—C14A—H14A	119.7	Na1A^i^—O4W—H4WB	139.0
C12A—C15A—H15A	109.5	Na1B—O4W—Na1A^i^	82.42 (7)
C12A—C15A—H15B	109.5	Na1B—O4W—H4WA	117.6
C12A—C15A—H15C	109.5	Na1B—O4W—H4WB	105.9
H15A—C15A—H15B	109.5	H4WA—O4W—H4WB	93.5
			
Na1A^i^—O1A—C1A—O2A	21.2 (3)	C15A—C12A—C13A—C14A	−178.7 (3)
Na1A^i^—O1A—C1A—C2A	−155.9 (2)	Na1B—O1A—C1A—Na1A^i^	−85.19 (19)
Na1A—O2A—C1A—Na1A^i^	−53.7 (6)	Na1B—O1A—C1A—O2A	−64.0 (4)
Na1A^i^—O2A—C1A—O1A	−19.5 (3)	Na1B—O1A—C1A—C2A	119.0 (2)
Na1A—O2A—C1A—O1A	−73.2 (8)	Na1B—O1B—C1B—O2B	40.8 (4)
Na1A^i^—O2A—C1A—C2A	157.4 (2)	Na1B—O1B—C1B—C2B	−141.7 (2)
Na1A—O2A—C1A—C2A	103.7 (7)	O1B—C1B—C2B—C3B	170.7 (3)
Na1A^i^—C1A—C2A—C3A	120.9 (5)	O2B—C1B—C2B—C3B	−11.7 (4)
O1A—C1A—C2A—C3A	−167.4 (3)	O3B—C8B—C9B—C10B	144.1 (3)
O2A—C1A—C2A—C3A	15.5 (4)	O3B—C8B—C9B—C14B	−34.1 (4)
O3A—C8A—C9A—C10A	−142.0 (3)	N1B—C3B—C4B—C5B	2.0 (3)
O3A—C8A—C9A—C14A	34.9 (4)	N1B—C6B—C8B—O3B	−16.1 (5)
N1A—C3A—C4A—C5A	−0.8 (3)	N1B—C6B—C8B—C9B	166.3 (3)
N1A—C6A—C8A—O3A	15.4 (5)	C1B—C2B—C3B—N1B	71.0 (4)
N1A—C6A—C8A—C9A	−168.0 (3)	C1B—C2B—C3B—C4B	−105.8 (3)
C1A—C2A—C3A—N1A	−70.1 (4)	C2B—C3B—C4B—C5B	179.2 (3)
C1A—C2A—C3A—C4A	110.7 (3)	C3B—N1B—C6B—C5B	1.6 (3)
C2A—C3A—C4A—C5A	178.5 (3)	C3B—N1B—C6B—C8B	169.0 (3)
C3A—N1A—C6A—C5A	−1.0 (3)	C3B—C4B—C5B—C6B	−1.1 (4)
C3A—N1A—C6A—C8A	−168.7 (3)	C4B—C5B—C6B—N1B	−0.3 (3)
C3A—C4A—C5A—C6A	0.2 (3)	C4B—C5B—C6B—C8B	−167.0 (3)
C4A—C5A—C6A—N1A	0.5 (3)	C5B—C6B—C8B—O3B	148.6 (3)
C4A—C5A—C6A—C8A	167.7 (3)	C5B—C6B—C8B—C9B	−29.0 (4)
C5A—C6A—C8A—O3A	−149.7 (3)	C6B—N1B—C3B—C2B	−179.5 (3)
C5A—C6A—C8A—C9A	26.9 (4)	C6B—N1B—C3B—C4B	−2.2 (3)
C6A—N1A—C3A—C2A	−178.3 (2)	C6B—C8B—C9B—C10B	−38.2 (4)
C6A—N1A—C3A—C4A	1.1 (3)	C6B—C8B—C9B—C14B	143.6 (3)
C6A—C8A—C9A—C10A	41.3 (4)	C7B—N1B—C3B—C2B	6.3 (4)
C6A—C8A—C9A—C14A	−141.8 (3)	C7B—N1B—C3B—C4B	−176.4 (3)
C7A—N1A—C3A—C2A	−6.0 (4)	C7B—N1B—C6B—C5B	175.7 (3)
C7A—N1A—C3A—C4A	173.4 (2)	C7B—N1B—C6B—C8B	−16.9 (4)
C7A—N1A—C6A—C5A	−173.2 (2)	C8B—C9B—C10B—C11B	−180.0 (3)
C7A—N1A—C6A—C8A	19.0 (4)	C8B—C9B—C14B—C13B	−179.1 (3)
C8A—C9A—C10A—C11A	178.7 (3)	C9B—C10B—C11B—C12B	0.3 (5)
C8A—C9A—C14A—C13A	−179.9 (3)	C10B—C9B—C14B—C13B	2.7 (5)
C9A—C10A—C11A—C12A	−0.5 (5)	C10B—C11B—C12B—C13B	0.3 (5)
C10A—C9A—C14A—C13A	−2.8 (5)	C10B—C11B—C12B—C15B	−178.3 (3)
C10A—C11A—C12A—C13A	0.3 (5)	C11B—C12B—C13B—C14B	0.6 (5)
C10A—C11A—C12A—C15A	177.7 (3)	C12B—C13B—C14B—C9B	−2.1 (5)
C11A—C12A—C13A—C14A	−1.3 (5)	C14B—C9B—C10B—C11B	−1.8 (5)
C12A—C13A—C14A—C9A	2.7 (5)	C15B—C12B—C13B—C14B	179.1 (3)
C14A—C9A—C10A—C11A	1.8 (4)		

Symmetry codes: (i) −*x*+2, −*y*+1, −*z*+1; (ii) *x*+1, *y*, *z*; (iii) *x*−1, *y*, *z*.

### Hydrogen-bond geometry (Å, º)

**Table:**

*D*—H···*A*	*D*—H	H···*A*	*D*···*A*	*D*—H···*A*
O1*W*—H1*WA*···O3*A*	0.89	2.12	3.002 (3)	169
O1*W*—H1*WB*···O1*A*^ii^	0.89	2.00	2.665 (4)	130
O2*W*—H2*WA*···O2*B*^ii^	0.89	1.88	2.741 (3)	161
O2*W*—H2*WB*···O2*B*^iv^	0.89	2.13	3.019 (3)	172
O3*W*—H3*WA*···O3*B*^v^	0.89	2.16	2.961 (3)	150
O3*W*—H3*WB*···O1*B*^iv^	0.89	1.84	2.699 (4)	161
O4*W*—H4*WA*···O1*B*^iv^	0.89	2.19	2.894 (3)	136
O4*W*—H4*WB*···O3*W*^iv^	0.89	2.02	2.889 (3)	163

Symmetry codes: (ii) *x*+1, *y*, *z*; (iv) −*x*+1, −*y*, −*z*+1; (v) −*x*, −*y*, −*z*+1.
